# Multiple Myeloma Cell-Derived Exosomes: Implications on Tumorigenesis, Diagnosis, Prognosis and Therapeutic Strategies

**DOI:** 10.3390/cells10112865

**Published:** 2021-10-24

**Authors:** Alessandro Allegra, Mario Di Gioacchino, Alessandro Tonacci, Claudia Petrarca, Caterina Musolino, Sebastiano Gangemi

**Affiliations:** 1 Division of Hematology, Department of Human Pathology in Adulthood and Childhood “Gaetano Barresi”, University of Messina, 98125 Messina, Italy; cmusolino@unime.it; 2 Center for Advanced Studies and Technology, G. D’Annunzio University, 66100 Chieti, Italy; claudia.petrarca@unich.it; 3 Institute for Clinical Immunotherapy and Advanced Biological Treatments, 65100 Pescara, Italy; 4 National Research Council of Italy (IFC-CNR), Clinical Physiology Institute, 56124 Pisa, Italy; atonacci@ifc.cnr.it; 5 Department of Medicine and Science of Ageing, G. D’Annunzio University, 66100 Chieti, Italy; 6 Department of Clinical and Experimental Medicine, Unit and School of Allergy and Clinical Immunology, University of Messina, 98125 Messina, Italy; gangemis@unime.it

**Keywords:** multiple myeloma, exosome, extracellular vesicles, miRNA, microenvironment, immune response, angiogenesis, osteoclast, chemoresistance

## Abstract

Multiple myeloma (MM) is a hematological disease that is still not curable. The bone marrow milieu, with cellular and non-cellular elements, participate in the creation of a pro-tumoral environment enhancing growth and survival of MM plasma cells. Exosomes are vesicles oscillating in dimension between 50 nm and 100 nm in size that can be released by various cells and contribute to the pathogenesis and progression of MM. Exosomes enclose proteins, cytokines, lipids, microRNAs, long noncoding RNAs, and circular RNAs able to regulate interactions between MM plasma cells and adjacent cells. Through exosomes, mesenchymal stem cells confer chemoresistance to MM cells, while myeloma cells promote angiogenesis, influence immune response, cause bone lesions, and have an impact on the outcome of MM patients. In this review, we analyze the role played by exosomes in the progression of monoclonal gammopathies and the effects on the proliferation of neoplastic plasma cells, and discuss the possible employment of exosomes as potential targets for the treatment of MM patients.

## 1. Introduction

### Overview of Exosomes

The intercellular interactions between hematological tumors and the bone marrow (BM) milieu are due to soluble components and to cell-to-cell connections. The three-dimensional positioning of multiple myeloma (MM) cells within the BM has an effect on their comportment. Partially, this is owed to the sorts and amounts of substances the neoplastic cells are subjected to, which are produced by their adjacent cells.

The element of the secretome that is acquiring increasing interest are the extracellular vesicles (EVs) and the exosomes. Exosomes are vesicles originated from cells oscillating in dimension between 50 nm and 100 nm in length with a denseness between 1.13 g/mL and 1.19 g/mL [1]. Corresponding to the content, dimensions, and mechanism by which they are generated, they can be separated into diverse subcategories [2,3], comprising exosomes, microvesicles and apoptotic bodies. The lengths of microvesicles (200–1000 nm) and apoptotic bodies (800–5000 nm) are greater than those of exosomes (30–150 nm) [2].

Exosomes are produced by the cells via merging of the multivesicular body (MVB) with the cell membrane. Other EVs, such as microvesicles, are generated by a direct blossoming from the plasma membrane. However, as none of the currently obtainable EV separation techniques can distinguish between true exosomes originated from the endosomal mechanism, or other small EVs, there is an agreement to just call them ‘small EVs’.

Small EVs or exosomes are constituted of proteins, DNA and RNA, and some which are cell-type specific.

Proteins of exosomes comprise costimulatory molecules such as CD86, antigen presenting proteins such as MHC-I and MHC-II, membrane fusion proteins such as annexins, intercellular adhesion molecule 1, integrins, tetraspanins, multivesicular body formation proteins such as Alix and Tsg10, and transmembrane molecules. These proteins are plentiful on the surfaces of exosomes, while other substances such as cytoskeleton proteins, heat shock proteins, and several types of enzymes such as glucose 6 and pyruvate kinase are present inside exosomes [3]. Lipids present in exosomes include cholesterol, ceramide, and sphingolipids, which are usually on the surfaces of exosomes. Among the most important molecules from the point of view of biological activity are RNAs (mRNAs), microRNAs (miRNAs) and other non-coding RNAs such as long non-coding RNAs and circular RNAs [4]. Baglio, et al. have established that BM mesenchymal stromal cell (MSC)-originated exosomes have a great content of tRNAs, with more than 35% of total small RNAs, while mature miRNAs represent only 2–5% of them [5]. Contrarily, from serum circulating miRNAs, exosomal miRNAs are rather stable in the circulation, owed to the protecting action of the exosome vesicles on degradation by RNases [6,7].

Once discharged from the MM cell, EVs can reach several targets; they can liberate their content in the neighboring interstitial space, or they can be taken by other cells at short or long distances. Cellular absorption is the securest method for EVs, as they seem to have a brief half-life in circulating blood, where it does not surpass two minutes before being reallocated into cells [8].

The systems by which exosomes are taken by cells comprise phagocytosis, clathrin- or caveolin-mediated endocytosis, micropinocytosis, and lipid raft–mediated endocytosis [9]. The employ of proteinase K decreases exosomes uptake by cells, so this suggests that proteins are essential for this procedure [10]. Tetraspanins have been reported to have a crucial effect in exosomal absorption in several type of cells. Other central proteins for exosomal absorption are lectins, integrins, and immunoglobulins.

All those different systems will allow for the accessing of the EVs to the intracellular environment, where they leave their bioactive loads [9]. RNA is placed in the endoplasmic reticulum in which it exercises its effects, while vacant EVs combine with lysosomes for degradation [11].

## 2. Exosomes and Multiple Myeloma

Multiple myeloma (MM) is characterized by the proliferation of clonal plasma cells into the BM, which generate monoclonal immunoglobulin. Despite improvements in the treatment of MM due to the discovering of novel drugs, most patients with MM relapse [12,13,14,15,16].

Exosomes can control biological communication and biological reactions [17,18]. In the context of MM, this guarantees unceasing interaction between MM cells and cells of the BM milieu, sustaining MM tumorigenesis by supporting immunosuppressive actions, angiogenesis, osteolysis and drug resistance. Furthermore, exosomes seem to have an effect in the genesis of cardiac damage, while in the onset of peripheral neuropathy they might have a prognostic role. Finally, exosomes have clear possibilities to be used as markers for initial prognostication of gammopathy progression [19,20,21].

**Figure 1:**
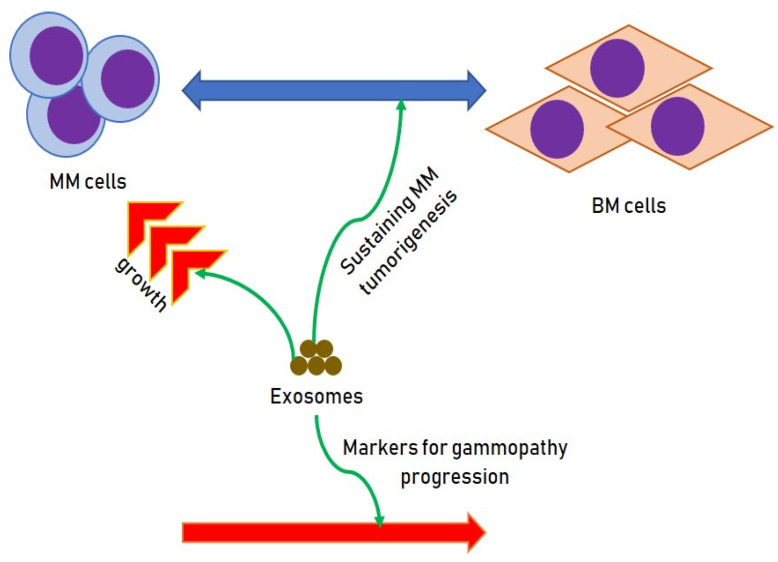
Effects of exosomes on MM cells.

Several experimentss have demonstrated that various components enclosed in exosomes can augment the growth of MM plasma cells (Figure 1). Principally non-coding genetic material such as miRNAs can transfer material able of augmenting the growth of clonal cells.

miRNAs are short non-coding RNA able to regulate protein expression via their targeting and silencing of complementary mRNA sequences. miRNAs can operate as cancer suppressor genes or as oncogenes, thereby influencing the survival of tumor subjects [22]. MiRNome expression in altered in BM and in the peripheral compartment of MM subjects [23,24] (Table 1).

As far the correlation between miRNAs enclosed in exosomes and MM, PIWI-interacting RNA (piRNA) is a sort of non-coding single stranded RNA which has a crucial effect on genome expression. A study demonstrated that PIWI-interacting RNA-004800 (piR-004800) are augmented in both exosomes originated from MM subjects’ BM supernatant and cultured MM cells [25]. The expression amount of piR-004800 is associated with the stages of the international staging system in MM patients. Moreover, in MM cell lines, a reduction of piR-004800 provoked programmed cell death and autophagy. This effect was associated with in vitro and in vivo decrease of cell growth. As far the mechanism, numerous data demonstrated that the sphingosine-1-phosphate receptor (S1PR) signaling pathway has an essential role in MM cell growth. In this experiment, the authors established that the S1PR signaling pathway can control the PI3K/Akt/mTOR pathway via regulation of piR-004800 expression. Moreover, in MM cells, they proved that the decrease of piR-004800 augmented cell death. This effect phenocopies the action of FTY720 (Fingolimod), a substance derived from the fungal metabolite myriocin. Furthermore, they determined the mutual action between sphingosine-1-phosphate and piR-004800. The blockage of the sphingosine-1-phosphate receptor by FTY720 decreases the generation piR-004800. These findings sustain an oncogenic effect for piR-004800 in MM [25].

Earlier reports performed on serum of MM subjects established that serum miRNAs such as miRNA-21 are extremely expressed and operate as oncogenes (oncomiRs) [26]. In a study, authors evaluated the influences of OPM2 (a MM cell line) and exosomes (OPM2-exo) on controlling the growth, and they revealed the effect of miRNA-21 and miRNA-146a. They determined that OPM2-exo harbored great concentrations of miRNA-21 and miRNA-146a and that the OPM2-exo coculture considerably increased MSC growth. Furthermore, OPM2-exo provoked cancer-associated fibroblast (CAF) transformation of MSCs and IL-6 generation. The blockage of miRNA-21 or miRNA-146a decreased these actions [27].

miRNAs can alter the BM milieu changing the cytokine balance, and an altered production of cytokines induced by miRNAs contained in exosomes could favor the growth of myeloma cells. For instance, the above-mentioned EV-mirNA-146a can change the reciprocal relation between MM and stromal cells. A study executed on EV-miRNAs originated from MM cells and transported to MSCs demonstrated that, among the 19 EV-miRNAs appreciably altered, EV-miRNA-146a was the most greatly increased. miRNA-146a provoked an augmented production of numerous tumorigenic cytokines by MSCs, comprising IL-6, IL-8, IL-10, CXCL1, CCL-5, and MCP-1 in a NOTCH signaling-dependent mode. Because of this, alteration of the chemokine production augmented MM plasma cell survival [28].

Alongside the miRNAs, other non-coded genetic material contained in the exosomes consists of long non-coding RNAs (lncRNAs). These are RNA transcripts with more than 200 nucleotides that are not transformed into proteins. LINC00461 was greatly produced in MM, and MSC-originated exosomes stimulated MM cell growth via LINC00461. Knockdown of LINC00461 significantly decreased MM cell growth and provoked programmed cell death. Analyses demonstrated that LINC00461 lessened the inhibitory action of miRNA-15a/miRNA-16 on BCL-2. These data proved that LINC00461, a sponge for miRNA-15a/16, may be a target for therapeutic approaches [29].

Another different type of non-coded genetic material is constituted by Circular RNAs (circRNAs), endogenous non-coding RNAs that exhibit a closed circular configuration that are is broadly disseminated in human tissues.

Circ_0007841 was reported to be increased in BM-derived plasma cells of MM subjects and MM cells.

Exosomes discharged from MSCs augmented the growth of MM cells via circ_0007841. Several data proposed that circ_0007841 altered cell cycle and reduced the programmed cell death of MM cells [30].

As for the way via which circ_0007841 enhanced the advancement of MM, it was demonstrated that miRNA-338-3p is an objective of circ_0007841 in MM cells. Bromodomain-containing proteins (BRDs) distinguish acetylated lysine (KAc) residues on the N-terminal tails of histones to reorganize chromatin. In the normal proteome, there are several bromodomains classified into diverse groups, one of which is the Bromodomain and Extra C-terminal domain (BET) family. Bromodomain-containing protein 4 (BRD4), an element of the BET family proteins, engages transcriptional elongation factor complexes and aids RNA polymerase II-mediated transcription [31]. BRD4 can connect to miRNA-338-3p in MM cells and miRNA-338-3p perform an anti-MM effect. Circ_0007841 augmented the stimulus of PI3K/AKT signaling via miRNA-338-3p/BRD4 axis. Thus, Circ_0007841 operated as an oncomiR via sequestering miRNA-338-3p to increase the expression of BRD4 [32].

However, apart from the action as transmitters of molecular information, exosomes have been reported to have intrinsic biological effects.

Vardaki et al. discovered that caspase-3 is stimulated in L88 BM stroma cell–originated exosomes and recognized one of the substrata to be the protein Bcl-xL, a controller of apoptosis. Incubation of the exosomes with a substance able to block caspases inhibits the cleavage of Bcl-xL. Moreover, biochemical blockade of Bcl-xL with ABT737 or inhibition by employing the D61A and D76A Bcl-xL mutant causes a relevant reduction in the uptake of exosomes by hematopoietic malignant cells. These data indicate that the cleaved Bcl-xL is required for the absorption of exosomes by MM, provoking an augmented growth. So, Bcl-xL is an exosomal caspase-3 substrate essential for the absorption of exosomes by recipient cells [33].

### 2.1. Exosomes and MM Angiogenesis 

**Figure 2:**
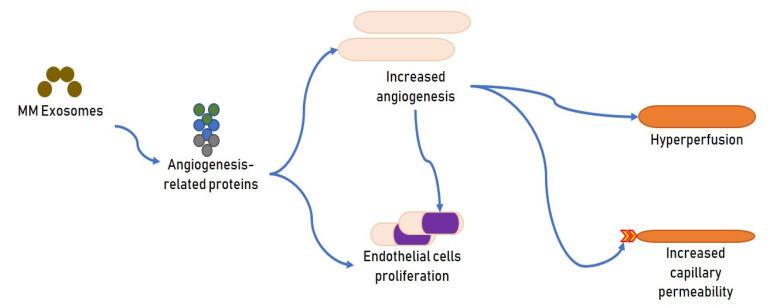
Effects of exosomes on angiogenesis.

Throughout MM advancement, the stimulation of the angiogenic activity is an essential moment for the establishment of the vascular niche, where diverse stromal elements and MM plasma cells cooperate and promote neoplastic proliferation [34]. Augmented angiogenesis is an invariable characteristic of MM progress and is central for its onset and diffusion [35]. VEGF-A, one of the principal components in the family of vascular endothelial growth factor, can stimulate angiogenesis and support MM cell growth [36]. Recently, angiogenesis has turned out to be one of the most relevant objectives in oncologic treatments and the anti-angiogenic substances have demonstrated to be very effective in MM patients. It is established that MM cells can act on endothelial cells via cell–cell contact or delivery of soluble substances able to create an advantageous milieu [37]. Alongside a direct effect on clonal cell growth, exosomes appear to modify the angiogenic dynamics that promote the progression of MM (Figure 2).

Murine MM exosomes transporting diverse angiogenesis-correlated proteins augmented angiogenesis and stimulated endothelial cell proliferation. Multiple pathways such as c-Jun N-terminal kinase, p53, and STAT3, were controlled by the exosomes in endothelial and BM stromal cells. Moreover, under hypoxia, MM cells produce components able to stimulate angiogenesis to modify this adverse micromilieu [38]. An augmented angiogenic effect relates to endothelial stimulation, hyperperfusion, and augmented capillary permeability [11]. Hypoxia signaling is controlled by hypoxia-inducible factor (HIF) as a central transcriptional regulator [39].

**Figure 3:**
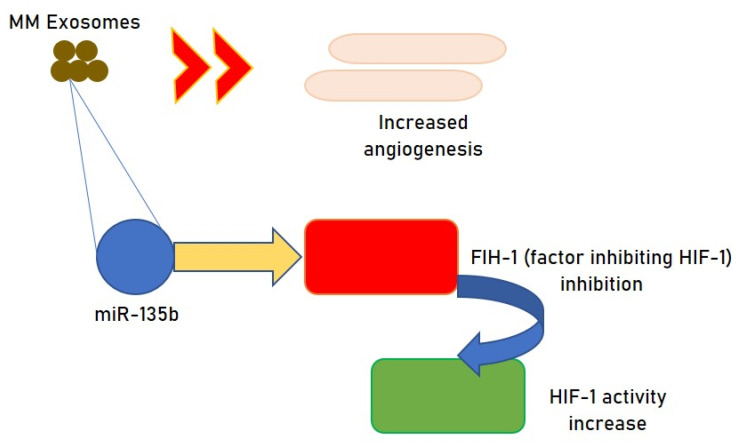
Hypoxic exosomes promote angiogenesis in MM.

Hypoxic environments are also reported to augment exosome generation. The BM is hypoxic per se, however, as MM cells grow the hypoxia augments, and exosomes produced by MM cells stimulate angiogenesis by modifying HIF-1 through miRNA-135b [40] (Figure 3). Moreover, exosomes produced under hypoxic stress can alter multiple diverse pathways and stimulate the movement of MM cells via the augmented generation of CXCL12/CXCR4/monocyte chemoattractant protein-1 axis [41].

Exosome miRNA analysis also ascertained a greater presence of miRNA-1305 in exosomes separated from hypoxic MM plasma cells than in those of normoxic cells. The survival of subjects with elevated exosomal miRNA-1305 was worse than it was in MM subjects with a small amount of exosomal miRNA-1305. In hypoxic MM cells, an augment of exosomal miRNA-1305 provoked a reduction of cellular miRNA-1305 and augmented stimulation of the miRNA-1305 downstream genes, such as *IGF1*, *MDM2* and *FGF2*, which are able to cause a stimulation of tumorigenic activity. Exosomal miRNA-1305 was also transported from MM cells to macrophages causing an MM-supporting M2-macrophage phenotype [42].

Finally, the antiangiogenic effects performed by exosomes derived from healthy controls seem to be exceptionally noteworthy as they could be employed for therapeutic objectives. In fact, exosomes originated from young BMSCs drastically blocked MM-caused angiogenesis. The exosomal miRNA expression outline was diverse between young and adult BMSCs. In any case, the antiangiogenic action of mature BMSCs was augmented by transfection of miRNA-340 that was especially present in exosomes originated from young BMSCs. An experimentation established that miRNA-340 blocked angiogenesis through the hepatocyte growth factor/c-MET (HGF/c-MET) signaling in endothelial cells. These findings offer novel information for exosome-based MM treatment by changes of BMSC-originated exosomes [43].

### 2.2. Exosomes and Immune Response in MM

An additional system by which exosomes could support the onset of MM and its progress is represented by the immunosuppressive actions they have. These actions could provoke both a decrease in immunosurveillance and an augmentation in the incidence of infectious complications that are often present in these patients.

An earlier experimentation established that tumor-originated exosomes (TEXs) tend to provoke immune depression and can inhibit the transformation of BM progenitor cells into dendritic cells (DCs) [44]. Moreover, exosomes can transport transforming growth factor β1 (TGF-β1) and alter the reaction of T cells to interleukin 2 (IL-2), permitting the conversion of lymphocytes into regulatory T cells (Tregs) instead of cytotoxic T cells [45]. Furthermore, TEXs support the stimulation and amassing of Treg cells [46]. Analogously, TEXs augment the delivery of IL-6 and prostaglandin E2 by MDSC, causing the development of a powerful immunosuppressive milieu in MM BM [47,48,49].

Numerous reports proved MM cell-originated exosomes have a strong effect on T lymphocyte immune activity. They support programmed cell death and block growth of HD-CD4+ T, block perforin of HD-CD8+ T, reduce programmed cell death and stimulate growth of HD-Treg, and decrease TGF-β delivery of MM-Treg [50].

Wang et al. evaluated the impact of BMSC-originated exosomes on the MM BM cells with an accent on MDSCs. MDSCs, as all immune cells that provoke immunosuppression, are closely implicated in controlling the resistance of tumor subjects to treatment and to prognosis [51,52].

An in vitro experimentation demonstrated that BMSC-originated exosomes increased the survival of Myeloid-derived suppressor cells (MDSCs), a phenotypically heterogeneous population that inhibits MM-specific T-cell responses. This effect is mediated by stimulating signal transducers and activators of transcription (STAT) 3 and STAT1 pathways and augmenting myeloid leukemia cell differentiation protein Mcl-1 [53]. The same group also reported that BMSC-derived exosomes stimulated MDSCs in vivo to augment their nitric oxide delivery, which participated t in the blocking of T cells [54]. They also demonstrated that MM-EXs also stimulated STAT3 in MDSCs to produce great amounts of arginase 1 and inducible nitric oxide synthase, which augmented T-cell suppression [55].

On the other hand, it has been established that natural killer (NK) cells have a central action in the progress of MM. NK cells can be stimulated in the early phase of MM and exert a cytotoxic action, destroying MM cells [56]. In a previous study we determined that MM-related NK cells are completely competent per se, but most of them miss their cytotoxic ability in the autologous set, probably because of the great expression of HLA class I molecules present on MM cells [57].

Numerous results have demonstrated that MM-originated small EVs decrease the cytotoxic capacity of NK cells against MM cells [58]. This powerful reduction of NK-cell ability is due to the ability of MM-originated EVs to provoke a decreased expression of most of the stimulating receptors recognized to be indispensable in determining NK cytotoxicity, comprising NKG2D, NKp46, and NKp30, [59].

A fascinating experiment evaluated the effects of MM-originated EVs on NK cells after genotoxic stress. Even though anti-tumor drugs were usually recognized to augment the immunogenic aptitude of tumor cells, this notion is no longer widely admitted. Vulpis et al. stated that when MM cells are treated with substances such as melphalan, a genotoxic molecule employed in the MM treatment, an increase in EV delivery is detected. MM-originated EVs absorbed by NK cells are able of increasing IFN generation, but not their cytotoxic capacity, via a system founded on the stimulation of the NF-kB pathway in a TLR2/HSP70-dependent way [60]. This is since after genotoxic stress, MM cells were reported to deliver great quantities of the metalloproteinase ADAM10, which is able to leak the NKG2D receptor ligand MIC. Once shed, NK receptor ligands become soluble and function to inhibit the receptors instead of stimulating them. Thus, NK cells miss their ability to produce the TNF alpha that is recognized to support their cytolytic effects [61]. A different MM-EV component that intensely reduces NK-cell activity is the MM-related antigen CD38. MM-originated EVs discharge great quantities of the soluble ectoenzyme CD38. This substance can transform nucleotides to adenosine, causing an inadequate immune response. Adenosine joins to the purinergic P2 receptors that is present on the membrane of immune cells to cause an insufficient response not only of NK cells, but of T-cells and dendritic cells, as well [62].

These findings have lately been proven in diverse experimental settings. L363 cells were exposed to docosahexaenoic acid (DHA) or eicosapentaenoic acid (EPA), two polyunsaturated omega-3 fatty acids with recognized anti-tumor actions, or were untreated. The discharged EXs (named as D-EX, E-EX, and C-EX) were employed to study NK cell functions. Myeloma EXs (C-EXs) considerably decreased NK cytotoxicity against K562 cells, while the cytotoxicity reduction was appreciably inferior in the D-EX- and E-EX-exposed NK cells with respect to the C-EX-exposed cells. The presence of the stimulating NK receptor NKG2D, and NK degranulation, after exposition to the EXs, were both modified. Nevertheless, C-EXs could augment IFN-γ delivery in NK cells, which was not appreciably modified by DHA/EPA exposition. This suggests a double action of MM EXs on NK cells activities and that MM EXs have both suppressive and stimulatory actions on diverse NK functions. Exposition of MM cells with EPA/DHA can decrease the suppressive actions of MM EXs while retaining their stimulatory actions. It is possible to hypothesize that DHA/EPA additions might be employed as an adjuvant therapy in MM patients [63].

### 2.3. Exosomes as Markers of MM Progression

Monoclonal immunoglobulin-correlated pathologies can evolve via several disease phases. There is presently no element forecasting the progression of MM from prodromal conditions. Serum exosomal miRNAs can be employed as new markers for MM and may be implicated in the progress of subjects with monoclonal gammopathies. In a study, the amount of serum exosome-originated miRNA-20a-5p, miRNA-103a-3p, and miRNA- 4505 were considerably diverse among patients with MM, smoldering multiple myeloma (SMM) and control subjects [64].

In a different study, three differentially expressed BMSCs-originated miRNAs (DEMs) were reported to discriminate MM from normal and MM-monoclonal gammopathy of undetermined significance (MGUS) controls in the GSE39571 dataset; one reduced and one augmented DEMs (hsa-miRNA-10a) could discern MM from normal and MM-MGUS controls in the GSE110271-GSE78865 merged dataset. Moreover, 11 reduced (hsa-miRNA-16) and 1 augmented DEMs were shared between GSE39571 and merged dataset when comparing MM with normal samples. The downstream genes were predicted for these 17 DEMs. *IGF1R* and *CCND1* were the more relevant genes and were controlled by hsa-miRNA-16. BMSCs-originated exosomal miRNA-10a and miRNA-16 may be implicated in MM progress by controlling the expression of genes such as *EPHA8* or *IGF1R/CCND1/CUL3/ELAVL1*. These exosomal miRNAs may be possible markers for prognostication of evolution and may be targets for novel therapeutic approaches [65].

A different analysis reported an alteration of exosomal lncRNA PRINS in MM vs. healthy controls. In particular, MM and MGUS subjects were differentiated from controls with a specificity of 83.3% and a sensitivity of 84.9%. Concentrations of PRINS were associated with classic MM chromosomal alterations, such as del(13)(q14), del(17)(p13), t(4;14), gain(1)(q21), and hyperdiploidy. This study proposes a potential diagnostic function for exosomal lncRNA PRINS in monoclonal gammopathies [66].

Positive results arose from the analysis of adenosine, too. This substance, a powerful immunosuppressor molecule generated by diverse cells in the BM of MM subjects, has an essential function in the tumor niche development and MM evolution [67]. It has been reported that MM-EXs are rich in ectoenzymes such as CD73, CD39, and CD38, which transform ATP and NAD+ (adenosine precursors) into adenosine [68]. These data, and the finding that BM concentrations of adenosine are greater in MM subjects with respect to MGUS/SMM subjects, suggest that exosomal adenosine might be employed as a marker both for the distinction of these diseases and for the differentiation of early or advanced stages in MM subjects [68].

Finally, Di Noto et al. described a new system for separating exosomes from MM, MGUS and healthy controls. The system is founded on the use of colloidal gold nanoplasmonics and surface plasmon resonance biosensing. It showed that MM subjects generate about a four-fold more exosomes than MGUS and normal subjects. Moreover, they demonstrated that only the MM-originated exosomes connect to heparin—an analog of heparan sulfate proteoglycans recognized to regulate exosome endocytosis with an elevated affinity binding. This system could be used to diagnose MM [69].

### 2.4. Exosomes and Organ Damage in MM

#### 2.4.1. Bone Disease

Exosomes appear to be able to induce the onset of organ damage and to influence the onset of bone disease, renal failure and heart damage in patients with amyloidosis.

At diagnosis, osteolytic lesions are present in about 60% of MM subjects. Moreover, almost every subject will exhibit a lytic lesion during the disease course, provoking an augmented morbidity and pain with a grave effect on the quality of life [70].

MM cells can model the BM milieu to support the tumour proliferation causing the niche remodeling and the occurrence of osteolytic bone disease [71]. Almost certainly this remodeling has a finalistic effect able to favor tumor progression. MM diffusion in the bone can employ this interaction system to alter the equilibrium between bone constructing and bone resorbing cells, which causes the delivery of bone produced substances promoting MM proliferation.

Sun et al. demonstrated that osteoclast (OCL)-originated exosomes are plentiful of ephrinA2, a protein which can communicate with osteoblasts (OBs) [72]. Furthermore, Deng et al. reported that MVs generated by OBs enclose RANKL protein and can transport it to OCL precursors promoting OCL generation [73]. In recent times, it has been stated that both OCL precursors and mature OCLs discharge EVs during osteoclastogenesis. EVs from OCL precursor stimulated osteoclastogenesis, while EVs from mature OCLs decreased the OCL amount in mouse BM cultures. The same experiments recognized a subgroup of EVs from mature OCLs enclosing great amounts of RANK that may competitively block the effect of RANK on OCL precursors by RANKL [74]. Moreover, two diverse reports suggested that exosomes produced by monocytes promoted the differentiation of BMSCs into OBs [75,76].

Other research has confirmed that the EV production could be a system by which MM cells can augment OCL activity. Interestingly, MM-originated exosomes are internalized by an OCL-like cell line sustaining the migration of OCL precursors through an augment of CXCR4 expression. MM-originated exosomes stimulated the expression of OCL markers such as TRAP, CTSK, and Matrix Metalloproteinases 9 in OCL-like cells. Furthermore, pre-OCLs exposed to MM-originated exosomes show a higher capacity to differentiate and reabsorb dentin substrate by blocking apoptotic dynamics. Analogous findings were achieved with exosomes separated from the plasma of MM subjects [77].

Moreover, MM cell-derived exosomes stimulated IL-6 production and reduced osteoblastic differentiation and mineralization of BMSCs. It was also reported that MM cell-originated exosomes provoke an augment in NF-kB and APE1 and a decrease in osteocalcin, runt-related transcription factor 2, and Osterix in BMSCs [78].

A report stated that EVs from the murine model of MM decreased OBs differentiation and their activity. In particular, the authors demonstrated that EVs transport the inhibitor of the Wnt/catenin pathway, Dickkopf-1, to OBs [79]. Moreover, it was described that exosomes augmented the expression of OC markers, such as cathepsin K, matrix metallopeptidase 9, and tartrate-resistant acid phosphatase [77].

The epidermal growth factor receptor (EGFR) is a glycoprotein that can be stimulated by a group of growth factors comprising amphiregulin (AREG). The EGFR system can regulate different processes such as growth and differentiation. Moreover, AREG has a central role in bone metabolism by influencing both OCs and OBs [80]. In fact, EGFR ligands can support OC growth by reducing in OBs the generation of OPG [81]. In addition to the EGFR, IL8, a cytokine recognized for its effects in supporting tumor angiogenesis [82] has been indicated as a stimulator of bone damage in MM bone disease [83,84].

A recent study confirmed that AREG can be released by MM exosomes and take part in MM-provoked osteoclastogenesis. In an experimentation, exosomes were separated from the medium of the MM1.S cell line and from BM samples of MM subjects, while cell line RAW264.7 and human CD14+ cells were employed as OC sources. They found that AREG was increased in MM exosomes and that exosomes-originated AREG caused the stimulation of EGFR in pre-OC, as demonstrated by the augment of mRNA of its target SNAIL. The use of blocking anti-AREG monoclonal antibodies (mAb) regressed this effect. Moreover, they stated the capacity of MM-originated AREG-enriched exosomes to enter MSCs inhibiting OB differentiation, augmenting MM cell adhesion and the delivery of the pro-osteoclastogenic cytokine IL8. Also in this case, anti-AREG mAb blocked the generation of IL8 by MSCs, proposing that direct and indirect mechanisms are involved in AREG-enriched exosomes osteoclastogenesis [85].

The statement that MM cell-originated exosomes provoked the stimulation of EGFR systems in MSCs and OC precursors implies the opportunity to employ drugs able to inhibit EGFR, such as gefitinib and erlotinib, and to alter the communication between MM plasma cells and the BM milieu to inhibit the onset of bone lesions. Previous in vitro studies have confirmed that erlotinib blocks lytic bone lesions in non-small lung cancer patients and that gefitinib decreases the capacity of MSCs to stimulate OC differentiation [86,87]. Another recent report confirmed the osteolytic actions of sEVs from the human JJN3 line when administered directly into the calvaria of NOD-SCID animals [88].

Employing the 5TGM1 murine model, authors demonstrated that 5TGM1 exosomes augmented OC functionality and inhibited OB differentiation and activity in vitro. Stopping exosome production employing the sphingomyelinase inhibitor GW4869 not only augmented cortical bone volume, but also it made sensitive the MM plasma cells to bortezomib, determining to a powerful anti-MM response when GW4869 and bortezomib were administered simultaneously. They studied the actions of 5TGM1 exosomes on the pre-osteoblast MC3T3-E1 line and detected caspase-mediated programmed cell death and a reduction in the presence of several genes correlated to OB differentiation. The consequences of 5TGM1 sEVs on vitality of MC3T3 cells were more evident than those of 5T33vt exosomes. This is due to the osteolytic potential of the cells of origin. Finally, they found that 5TGM1 exosomes provoked a reduction of Runx2 in MC3T3-E1 cells. These findings appear to be the consequence of an inhibition of the Wnt signaling system, as demonstrated by the eduction of whole and active β-catenin [19].

In the context of the relationship between exosomes and bone disease in MM, a fundamental role seems to be played by non-coding genetic material. An experimentation demonstrated that miRNA-214-containing exosomes produced by OCLs participate in the communication between OCLs and OBs. miRNA-214-exosomes can be transported into OBs via the ephrinA2/EphA2 system to inhibit OB functionality. The amounts of miRNA-214 were reportedly increased in exosomes and in serum from osteoporotic subjects versus non-osteoporotic subjects, proposing that it may be useful as a marker for bone loss [72].

In a different study, Raimondo et al. evaluated the relationship between the EV-derived osteogenic inhibition and MM-exosome content, focusing on miRNAs. They recognized a group of miRNAs significantly present in MM cell line- derived exosomes (MM1.S EVs) and in BM-exosomes derived from subjects affected by MM or SMM. Remarkably, they discovered that miRNA-129-5p, which affects several OBs differentiation markers, is increased in MM-EVs with respect to SMM-EVs, so they proposed a specific profile associated with a specific pathological condition. Moreover, they evidenced that miRNA-129-5p can be transferred to hMSCs by MM-EVs, and the augment of miRNA-129-5p amounts in hMSCs blocked the appearance of the transcription factor Sp1, a positive controller of osteoblastic differentiation, thereby recognizing miRNA-129-5p as a protagonist of vesicle-caused bone lesions [89].

Long noncoding RNAs (lncRNAs) are also powerful controllers of cell differentiation with cell-specificity that are produced by tumor cells through exosomes. In MM, the exosomal transport of the lncRNA RUNX2-AS1 particularly blocks the osteogenic differentiation ability of MSCs by inhibiting the controller of bone generation RUNX2 [90].

Furthermore, Li et al. confirmed these findings. RUNX2-AS1 was able to develop an RNA duplex with RUNX2 premRNA at overlapping regions, and this RNA reduced RUNX2 expression by decreasing the splicing efficacy, determining a reduced osteogenic capacity of MSCs. In experimental animal models, the use of an inhibitor of exosome production, GW4869, was able to prevent bone loss. Thus, exosomal lncRNA RUNX2-AS1 may be a possible target for the treatment of osteolytic lesions in MM [91]. Nevertheless, before lncRUNX2-AS1 can be employed as therapeutic target, confirmation of its transport in vivo necessitates to be confirmed. Moreover, dispensation of GW4869 in experimental animal models extensively alters exosome delivery from all cell sorts and can provoke the discharge of EV subpopulations budding from the plasma membrane [92].

#### 2.4.2. Exosomes and Renal Function

A report studied the possible correlation between exosomal miRNAs and clinical manifestations in MM subjects. The data established that the presence of miRNA-140-3p, miRNA-185-5p, miRNA-425-5p, let-7c-5p, and let-7d-5p in the exosomes of MM subjects were appreciably inferior with respect to those of normal subjects. Moreover, there were essential modifications in the clinical symptoms of MM, such as renal failure. The amounts of exosomal miRNAs were correlated to the concentrations of clinical feature-corelated markers, such as creatinine, IL-6, *β*2-microglobulin and *β*-CTX in serum [93].

#### 2.4.3. Exosomes and Heart Failure

Cardiac alterations are one of the most critical MM comorbidities and can provoke a serious cardiomyopathy and heart failure secondary to cardiac amyloidosis or anemia. Moreover, particular drugs employed for MM treatment can disturb cardiac function [94], and several studies proved that up to 50% of MM subjects have heart impairment [95].

Informatics investigation demonstrated that circRNAs with augmented might cause MM-correlated myocardial alteration. Furthermore, PCR findings established that circ-G042080 was copiously present in the serum exosomes of MM subjects. The expression amount of circ-G042080 was statistically associated with the MM-correlated heart failure. The myocardial damage might be due to a downstream miRNA/TLR4 axis. Experimental analyses proved that the circ-G042080/hsa-miRNA-4268/TLR4 axis might be present in H9C2 cells incubated with exosomes and might provoke abnormal autophagy. Exo-circRNAs might be a novel diagnostic biomarker of MM-correlated heart damage and might be a therapeutic target [96].

#### 2.4.4. Exosomes and Concomitant Pathologies in Patients with MM

The biological activity of endosomes could play a role in some pathological conditions often present in patients with MM such as thrombotic events and iatrogenic neuropathy.

Thrombosis is a well-recognized complication to MM [97], and the substances contained in the exosomes have a pro-coagulant activity and augment endothelial cell (EC) thrombogenicity, indicating their participation in MM-correlated thrombosis. Exosomes enclose great concentrations of angiogenic elements that influence mesenchymal and EC, cause cell growth through specific signal transductions.

Bortezomib-treated exosomes show decreased concentrations of angiogenic components, which reduce migration of MVs, reproducing the effectiveness of MM treatment [98].

Peripheral neuropathy (PN) is a consequence of MM or MM treatment which negatively influences MM subjects’ quality of life [99]. PN is caused by penetrating of M protein, compression phenomena by the tumor or by therapy-induced neurotoxicity. Several reports have established that almost 20% of MM subjects have PN at the beginning of the MM and about 75% have chemotherapy-caused PN (CIPN) [100].

An experimentation evaluated the relationship between serum exo-circRNAs and MM in MM-related PN [101]. A group of MM subjects and normal controls were studied. A correlation was searched between chr2:2744228-2744407+ and features of PN. 265 increased circRNAs and 787 decreased circRNAs, with a two-fold difference in expression level in MM patients versus normal subjects were evaluated. Informatics evaluation suggested that increased circRNAs had the aptitude to promote MM-correlated PN. Moreover, PCR confirmed the copious presence of chr2:2744228-2744407+ in the exosomes of MM subjects.

Chr2:2744228-2744407+ might cause MM correlated PN through the miRNAs and GRIN2B axis. An augment of chr2:2744228-2744407+ in the exosomes of MM patients might provoke the decrease of hsa-miRNA-6829-3p and increase of GRIN2B in the serum. The expression of chr 2:2744228-2744407+ was correlated with the clinical features of PN, suggesting that exo-circRNAs might be a new therapeutic target for MM correlated PN [101].

#### 2.4.5. Exosomes and Graft Versus Host Disease

Allogeneic hematopoietic stem cell transplantation (HSCT) is a therapeutic approach that can be evaluated in young subjects with grave MM. Nevertheless, transplant-correlated problems, such as acute and chronic graft-vs-host disease (GVHD) are a significant reason for mortality [102]. Lia et al. performed an experimentation on MM patients subjected to allogeneic HSCT to evaluate exosomal antigens as possible markers for acute GVHD. CD146 correlated with a 60% augmented risk of arising GVHD, while CD31 and CD140-a had a 40% and 60% decreased risk, respectively [103].

### 2.5. Exosomes and Prognosis

Recent data established the usefulness of exosomes in discovering relapse months before existent clinical analyses, underlining the reliability for exosomes in checking MM progression and evaluating minimal residual disease in MM subjects [104]. Furthermore, the evaluation of exosomes generated by MM subjects appear to be capable of assuring a prognostic assessment.

At this regard, an estimation of exosomal miRNAs separated from the MM subjects recognized 22 miRNAs that were reduced with respect to normal subjects. Among those, miRNA-18a and let-7b were appreciably correlated with overall survival (OS) and progression-free survival. Moreover, MM subjects with inferior exosomal miRNA-18a and let-7b concentrations were more frequently in an advanced stage of the ISSD and had a poor prognosis [105].

Similarly, MM-originated exosomes transporting CD138, a biomarker of mature plasma cells, were also found in the plasma of MM subjects, and their concentrations were associated with MM state and tumor burden. Subjects with aggressive MM had an important increase in serum EV-CD138 with respect to patients in partial or complete remission [104]. Moreover, employing exosomes proteomic profiling, it was possible identify phagocytic glycoprotein-1 (CD44) as a new biomarker that negatively correlated with OS. MM subjects with augmented risk of death presented augmented CD44 in their sera [106]. Therefore, exosomal-CD44 is another antigen that might be employed as a prognostic marker in MM.

A different approach is that constituted by the phage display technique, a modality often used for the study of hematological diseases [107]. Iaccino et al. evaluated the generation of MM-related exosomes in the murine 5T33MM MM model as markers of tumor proliferation. To this purpose, they chose Id-peptides by selecting a phage display library employing as bait the Ig-BCR expressed by 5T33MM cells. The FITC-conjugated Id-peptides identified the MM-related exosomes in the serum of 5T33MM engrafted mice, amounts of which are related with MM progression at an earlier time with respect to serum paraprotein. These findings suggest that Id-peptide-based evaluation of MM-released exosomes may be an extremely sensitive diagnostic technique for assessment of MM progression [108].

### 2.6. Exosomes and Chemoresistance

Multiple factors causing proteasome inhibitor (PI) resistance have been considered, such as genetic mutations and BM microenvironment changes [109]. However, conditions able to cause drug resistance in MM are not well recognized. In fact, in addition to the changes of myeloma cells in response to chemotherapy, several data suggest that the interactions between the MM plasma cells and the cellular elements of the contiguous BM microenvironment provoke the delivery of pro-survival signals causing drug resistance. This type of drug resistance, named “cell adhesion-mediated drug resistance” (CAM-DR), is regarded as the most important mechanism able to cause the escape of MM cells from therapeutic effects [110,111].

Several results appear to corroborate the chance that exosomes discharged in the BM milieu of MM subjects may participate in the establishment of a condition of chemoresistance. In research, the medical reports of hospitalized MM subjects, who were receiving new drug-based treatments, were studied. An exosomal RNA analysis was performed, and the exosome-derived miRNA profile for foreseeing drug resistance was evaluated employing a microarray. In the study, 204 MM subjects with drug resistance (DR) rates of 36.5%, 73.1% and 81.8% in the bortezomib (Bz), thalidomide and lenalidomide groups were included. An increased risk for predicting de novo DR was 1q21 gain. In the subjects resistant to Bz, an increased level of exosomes was found with a reduction of exosomal miRNA-15a-5p, miRNA-16-5p, miRNA-20a-5p, and miRNA-17-5p [112].

Similarly, an exosome-related miRNA expression profile executed on MM subjects resistant to Bz showed that exosomal miRNA-15a, miRNA-16, miRNA-17 and miRNA-20a were intensely reduced, and were correlated with drug resistance [74]. Since a great part of MM subjects have primitive or secondary resistance to Bz during the treatment, the above-mentioned profile of exosome-miRNAs could be employed as prognostic markers for Bz resistance and should be used to accomplish a personalized treatment [28].

Furthermore, some experimentations have attempted to elucidate the processes by which exosomes can cause chemoresistance. Xu et al. established that lncRNA PSMA3 and PSMA3-AS1 in MSCs could be enclosed into exosomes and transported to MM plasma cells, thus provoking proteasome inhibitor resistance [113]. PSMA3-AS1 could develop an RNA duplex with pre-PSMA3, which augment PSMA3 expression by enhancing its stability. In experimental animal models, administration of siPSMA3-AS1 was reported to be efficacious in modifying carfilzomib sensitivity. Finally, circulating exosomal PSMA3 and PSMA3-AS1 were correlated with PFS and OS in MM patients [113].

A different analysis described an augment in acid sphingomyelinase (ASM) presence in MM cell lines exposed to melphalan or Bz, and their exosomes. Exosomes with a great ASM content were capable of transmitting the drug-resistant phenotype to chemosensitive cells, thereby proposing an MM-protective action for ASM. Furthermore, the blockade of ASM by amitriptyline augmented drug sensitivity in MM cells was observed. These findings postulate a rationale to incorporate drugs able to target ASM in combination with traditional MM treatments [114].

Other non-lipid substances may be important in establishing chemoresistance. A study established a relevant effect of the chondroitin sulfate proteoglycan serglycin in controlling the protein cargo of MM plasma cell-originated exosomes. Earlier experimentations have demonstrated that serglycin operates essentially in the storing of basically charged elements within the intracellular vesicles through serglycin’s densely clustered, negatively charged glycosaminoglycan chains. Serglycin was found in exosomes derived from MM cell lines and from MM subjects. Exosomes originating from serglycin-knockdown cells, but not from normal cells, were deficient in several types of proteins that are essential for determining numerous cellular processes. For instance, exosomes from serglycin-knockdown cells were not able to determine an aggressive phenotype in MM plasma cells and were incapable of stimulating the migration of macrophages. These results demonstrate that serglycin has a relevant effect in supporting the protein cargo in MM-originated exosomes and proposes that targeting serglycin may modify the effect of these exosomes on MM progression [115].

Finally, the circRNA circMYC (hsa_circ_0085533) is originated from the *MYC* gene and has been stated to augment the growth of tumor cells [116,117]. The amount of the serum exosomal circMYC was appreciably augmented in MM subjects with respect to normal controls. Furthermore, the concentrations of circMYC in circulating exosomes was considerably higher in bortezomib-resistant patients that in non-resistant subjects. The levels of exosomal circMYC was related with the deletion of 17p, t(4;14) and with the stage of disease. A great exosomal circMYC concentration was an independent marker of bad prognosis in MM subjects, and patients with greater exosome circMYC levels had superior relapse percentages and greater mortality frequency. Contrariwise, the OS and PFS of MM subjects with increased exosomal circMYC expression were inferior to those of MM subjects with reduced exosomal circMYC levels [118].

A further system able to induce chemoresistance could be Heparanase. It is an endo-β-D-glucuronidase that operates cleaving heparan sulfate chains. It has several targets able to augment the expression and function of protease, growth factors, and RANKL that stimulate MM proliferation, diffusion, and the onset of bone lesions [119] via BM milieu modification and increasing angiogenesis [120].

An experiment explained the role of heparinase that revealed a direct correlation between Bz sensitivity, heparanase, and miRNA-1252-5p expression. An increased expression of miRNA-1252-5p appreciably decreased heparanase expression and function in MM cells, and the greater amount of miRNA-1252-5p was related to a decreased cell viability and a greater sensitivity to Bz. Furthermore, exosomes transporting miRNA-1252-5p augmented MM cells’ sensitivity to Bz therapy. These findings demonstrated that the employing of exosomes transporting containing miRNA-1252-5p might be a possible new Bz sensitization system in MM cells [121].

An interesting aspect related to the study of exosomes in MM is constituted by the variations induced by chemotherapy on the structure and function of the exosomes. When MM plasma were treated with drugs such as Bz, carfilzomib or melphalan, exosome generation by the cells was significantly augmented [122]. These chemotherapy-changed exosomes, named ‘chemoexosomes’ have a proteome structure different from that of plasma cells not treated with drugs comprising the above-mentioned augmentation in the amount of heparanase present as exosome cargo. The chemoexosome heparanase was not located in the chemoexosome but was found on the exosome membrane where it was capable of destroying the heparan sulfate of the extracellular matrix. Chemoexosomes transported their heparanase to MM cells augmenting their activity and causing a stimulation of ERK signaling and an augment in discharging the syndecan-1 proteoglycan. Moreover, chemoexosomes-enhanced secretion of TNF-α, by macrophages, and this cytokine is an essential MM growth factor. Finally, chemoexosomes augmented macrophage diffusion, and this action was inhibited by a monoclonal antibody, H1023, that blocks heparanase [122]. These findings suggest that anti-myeloma treatment stimulates a relevant production of exosomes having a great amount of heparanase that modifies the extracellular matrix and changes the BM microenvironment contributing to the onset of chemoresistance and patient relapse. Therefore, it is possible that changing the delivery or the absorption of exosomes could contrast the onset of chemoresistance.

Blocking endocytosis reduces the exosome-caused decrease of chemosensitivity to Bz, and thereby augments its anti-MM activities. In this regard, numerous experiments have been conducted. Small exosomes originated from BMSC were isolated from MM cells and were able to augment MM cell proliferation and decrease chemosensitivity to Bz. The employment of endocytosis inhibitors targeting molecules such as tyrosine kinase, heparin sulphate proteoglycans actin, or phosphoinositide 3-kinases decreased MM cell internalization of BMSC-originated exosomes. A different approach involved the use of shRNA-mediated knockdown of endocytosis-related proteins such as flotillin-1, caveolin-1, and clathrin heavy chain. This technique was able to reduce exosome-absorption in MM plasma cells. Finally, an endocytosis blocker able to target dynamin-2 reduced the absorption of exosomes by MM plasma cells ex vivo and increased the anti-MM actions of Bz in vitro and in an experimental animal model [123].

### 2.7. Exosomes as a Target Therapy in MM

Exosomes release signals to several types of cells and could so be manipulated as a novel therapeutic instrument. TRAIL-armed exosomes can stimulate programmed cell death in tumor cells and regulate tumor progression in vivo. Rivoltini et al. evaluated the capacity of membrane equipped exosomes to release signals able to augment programmed cell death in K562 cells (chronic myelogenous leukemia) and cause a reduced proliferation in diverse tumor models in vivo and in vitro [124].

Interfering with the angiogenic dynamics caused by exosomes could have a therapeutic effect in MM. Histone deacetylases (HDACs) are therapeutic objectives in MM, and it was demonstrated that HDAC3 blockade reduces MM growth. Pharmacologic block, knock-out (KO), and knock-down (KD) of HDAC3 in BMSCs causes a reduction of MM plasma cell growth. A study established a correlation between modifications in exosomes and exosomal miRNA, HDACs inhibition and anti-MM activity. The authors demonstrated that HDAC3-KD in BM endothelial cells reduces neoangiogenesis [125].

Ceramide, a sphingolipid, can cause the blocking of proliferation, death, and senescence in tumor cells [126]. However, the ceramide system is correlated with the activity of exosomes. Exosomes discharged from MM plasma cells exposed to C6-ceramide (C6-cer) (ExoC6-cer) appreciably reduced the growth, diffusion, and generation of ECs. A study established that the concentration of miRNA-29b was augmented in ECs exposed by ExoC6-cer, wheals mRNA expression of VEGFA, Akt3, and PI3K were reduced in ECs, suggesting the presence of an Akt system. Moreover, a reduction of miRNA-29b by inhibitor dispensation could avoid the ExoC6-cer-caused cell growth and angiogenesis of ECs associated with the augmented production of Akt3, PI3K and VEGFA [127].

Apart from changing exosome delivery, altering correlation between exosomes and adjacent cells to avoid exosome absorption also is a therapeutic opportunity. Purushothaman et al. established that heparan sulfate has a multiple action in controlling exosome-cell relationship, taking fibronectin on exosomes, and operating as a receptor for fibronectin on target cells. The binding of the fibronectin of exosomes to target cells can stimulate systems like p38 and pERK and modify the presence of DKK-1 and MMP-9, two substances able to intervene in MM progression [128]. Moreover, they demonstrated that elimination of heparan sulfate employing bacterial heparitinase or utilizing antibody specific for the Hep-II heparin-binding domain of fibronectin significantly blocks the exosome-target cell relationship [128]. The heparin-derived molecule Roneparstat blocked the effects of exosomes with a good safety profile in a phase 1 clinical trial (NCT01764880) [129].

Even some of the drugs generally used in the treatment of MM could perhaps act at least in part through an action on the exosomes, stimulating the immune response or modulating immune surveillance. In fact, some reports demonstrated that exosomes presenting great concentrations of heat shock protein 70 (HSP70) could stimulate NK cell responses [130]. Moreover, senescence is the effect of a cellular program triggered by genotoxic, or oncogenic stress, in which cells topped their cycle but continue to be metabolically active and produce numerous soluble components. This phenomenon is recognized as the senescence-associated secretory phenotype (SASP), which regulates several cellular responses, comprising variation of tumor immune surveillance. Several data clarified the effects of exosomes in regulating the cell-to-cell diffusion of senescence signs, proposing that exosomes may operate as a controller of the SASP [131]. Exposure of MM cells with sublethal dosages of genotoxic substances provokes senescence and causes an augmented NK cell recognition. An experimentation stated that melphalan- and doxorubicin-exposed senescent cells show augmented expression of exosomal IL-15, a cytokine implicated in NK cell growth and stimulation. However, IL15 was evident as a soluble cytokine only in vivo, so suggesting a role of IL-15 in the BM MM milieu. The augmented IL-15 was associated by augmented expression of the IL15/IL15RA on the surface of senescent MM plasma cells, permitting the functional trans-presentation of this molecule to adjacent NK cells, which subsequently underwent stimulation and growth [132]. These findings suggest that manipulating these exosome-induced processes might permit the employ of novel senescence-based cancer therapies.

## 3. Conclusions and Future Perspectives

Exosomes are essential intermediaries of cell–cell relationships at short and long distances. In hematological malignancies, via the discharge of exosomes, tumor cells interrelate with a huge number of cells, comprising cells of the tumor BM milieu that enhance disease progress by causing a reduction of immune response and provoking drug resistance. Exosomes released by tumor and BM cells enclose a wide range of tumor-specific molecules, comprising oncogenes and onco-miRNAs. The employment of appropriate antagoMirs able of modifying the function of the non-coding genetic material contained in the exosomes could constitute a new approach for the therapy of MM. Moreover, thinking through the essential action performed by exosomes in the onset of bone lesions disease, aiming exosomes delivery or absorption could consequently improve bone disease and prognosis [133].

The advantages of exosome treatment are its small toxicity, biological barrier penetrability, stability, and biocompatibility [134]. A different advantage of exosomes in hematological malignancies is the possibility to use them as a potential non-invasive “liquid biopsy”, given their great copiousness in biofluids and their capacity to defend their content from nuclease and protease degradation. Liquid biopsies are tests that offer an alternative system of disease diagnosis and staging, monitoring disease progression and response to therapy [135].

Furthermore, in the near future, new techniques may be used to study exosomes and the substances they contain. Raman spectroscopy and its improved version, surface-enhanced Raman spectroscopy (SERS), are methods employed for the study of hematological neoplasms [136,137,138]. Raman spectroscopy and SERS have also been employed for differentiating MGUS, aMM, and sMM patient-originated EXs. Russo et al. have established the ability of Raman spectroscopy for differentiating EXs along the passage from MGUS to aMM and sMM, thus offering advantageous clinical suggestions for patient management. The combined use of Raman spectroscopy with the adopted multivariate analysis (PCA) has efficaciously divided subjects belonging to these three groups. Remarkably, while sMM subjects are undoubtedly divided from aMM and MGUS, these latter clusters have more comparable although still different profiles. The employed SERS devices, founded on random nanostructures, have demonstrated good sensitivity, but further studies are necessary in order to obtain reliable and reproducible results [139].

Furthermore, the utilization of exosomes could be advantageous for the realization of an adequate vaccinotherapy of MM, allowing the overcoming of the numerous obstacles that still make it impracticable [140]. According to a study by Xie et al., vaccines developed from exosomes were efficacious for MM. In their studies, exosomes originated from MM cells were employed to promote anti-MM immune response and induce prophylactic immunity in MM cell lines. Multiple myeloma special antigen-1, a membrane protein, is exclusively present in MM cells. Employing its epitope, a vaccine named SLSLLTIYV stimulated a powerful cytotoxic T lymphocyte response in vitro. Moreover, MM special antigen-1-derived epitopes could join to Dickkopf-1 to generate a multi-epitope peptide vaccine that markedly enhanced the survival of immune deficient mice affected by MM with respect to a single-epitope vaccine. This approach reduced the tumor burden and decreased the number of bone lesions [141,142].

Finally, exosomes could be used as nanocarriers for drug delivery. The membranes of exosomes may merge with the membranes of the adjacent cells, so enclosed drugs can be transported to the target cells. In this context, another advantage is that the membranes of exosomes can defend the therapeutic substances from fast clearance by the mononuclear phagocyte system, thereby protracting the exposition time. Moreover, as exosomes are endogenous components, they present a biocompatibility and biodistribution like that of liposomes and may be the perfect biological nanocarrier for drug transport [143,144,145].

However, there are still problems in the separation of exosomes and their production. For this reason, several attempts have been performed to generate exosome mimetics (EMs), which are structures artificially separated from cells. The dimensions and compositions of EMs are like those of exosomes. In a study by Jiang et al. [146], a human U937 monocytic cell line and the mouse Raw264.7 macrophage cell line were pressed out via polycarbonate membranes in the presence of doxorubicin, generating doxorubicin-loaded EMs (DOX-EMs). The authors demonstrated that DOX-Ems decreased tumor proliferation in animals carrying a transplanted mouse colon cancer cell line. Moreover, they confronted the antineoplastic effects of DOX-EMs and doxorubicin-loaded exosomes, establishing that the DOX-EMs had analogous anti-tumor function with respect to doxorubicin-loaded exosomes.

The more recent molecules affecting MM cells generated to date are monoclonal antibodies (mAb), comprising anti-CD138 mAb, anti-CD38 mAb, anti-SLAMF7 mAb, and anti-BCMA mAb which have been employed in targeted treatment for MM patients [147,148,149,150]. Based on the study presented above, the inserting of doxorubicin in exosomes, or EMs, and the variation of exosomes with molecules such as anti-myeloma mAbs, may be a novel approach for the treatment of MM.

Overall, exosomes give the possibility to both enhance our knowledge of the molecular systems of MM pathogenesis and to offer a novel, efficacious therapeutic strategy in MM patients [21]. A great deal of evidence has demonstrated that interferences with the EX-mediated interactions within the tumor niche can augment the therapeutic effectiveness of routine chemotherapeutic drugs, overwhelm drug resistance, and avoid the onset of several MM-associated complications such as osteolytic bone lesions and renal failure.

## Figures and Tables

**Table 1:** Main effects of non-coding genetic materials on multiple myeloma.

Non-Coding RNA	Status in MM	Effect	Mechanism	Type of Study	Ref.
piR-004800	Augmented	Reduced apoptosis and autophagy	PI3K/Akt/mTOR signaling	In vitro	25
miRNA-21	Augmented	Effect on BM milieu	IL-6 generation, CAF transformation	In vitro	27
miRNA-146a	Augmented	IL-6, IL-8, IL-10, CXCL1, CCL-5, MCP-1 delivery, CAF transformation	NOTCH signaling	In vitro	27, 28
miRNA-135b	Augmented	Increased angiogenesis	Effect on HIF-1	In vitro	41
miRNA-1305	Augmented	Reduction of cellular miRNA-1305. Induction of M2-macrophage phenotype	Increased expression of *IGF1*, *MDM2* and *FGF2*	In vitro and in vivo	42
miRNA-340	Originated by Bone Marrow Stromal Cells	Reduction of angiogenesis	Effect on hepatocyte growth factor/c-MET signaling		43
miRNA-20a-5p, miRNA-103a-3p, miRNA- 4505	Augmented in monoclonal gammopathy	Effect on gammopathy progression	Not known	In vivo	64
miRNA-10a and miRNA-16	BMSCs-originated exosome	Effect on gammopathy progression	Increased expression of *EPHA8* or *IGF1R/CCND1/CUL3/ELAVL1*	In vitro	65
miRNA214	Osteoclast originated exosome	Effect on bone lesions	Inhibition of osteoblast functionality	In vitro	72
miRNA 129-5p	Augmented	Effect on osteoblastic differentiation	Effect on transcription factor Sp1	In vitro	89
miRNA-140-3p, miRNA-185-5p, miRNA-425-5p, let-7c-5p, and let-7d-5p	Reduced	Effect on kidney function	Not known	In vitro	93
miRNA-15a-5p, miRNA-16-5p, miRNA-20a-5p, and miRNA-17-5p	Reduced	Bortezomib chemoresistance	Not known	In vivo	112
miRNA-15a, miRNA-16, miRNA-17 and miRNA-20a,	Reduced	Chemoresistance	Not known	In vitro	74
LncRNA00461	Augmented	Increased cell proliferation, reduced apoptosis	Inhibitory action of miRNA-15a/miRNA-16 on BCL-2	In vitro	29
LncRNA PRINS	Augmented	Effect on gammopathy progression	Genetic mutations?	In vivo	66
LncRNA RUNX2-AS1	Augmented	Inhibition of osteogenic differentiation of MSCs	Inhibition of RUNX2	In vitro	90
LncRNA PSMA3 and PSMA3-AS1	MSCs derived	Proteasome inhibitor resistance	Development of an RNA duplex with pre-PSMA3	In vitro and in vivo	113
Circ_0007841	Augmented	Altered cell cycle and reduced programmed cell death	Effect on PI3K/AKT signaling via miRNA-338-3p/BRD4 axis	In vitro	30, 32
Circ-G042080	Augmented	Myocardial damage	Effect on miRNA/TLR4 axis	In vitro	96
CircMYC (hsa_circ_0085533)	Augmented	Bortezomib resistance		In vivo	116–118

## Data Availability

Not applicable.
